# Dysgerminoma with Estrogen-Producing Functioning Stroma Presenting Precocious Puberty

**DOI:** 10.1155/2021/5545645

**Published:** 2021-04-22

**Authors:** Shunsuke Nagase, Kanako Ogura, Karin Ashizawa, Nana Nakazawa-Tanaka, Masahiko Urao, Masaharu Fukunaga, Yuto Yamazaki, Hironobu Sasano, Toshiharu Matsumoto

**Affiliations:** ^1^Department of Diagnostic Pathology, Juntendo University Nerima Hospital, Tokyo, Japan; ^2^Department of Pediatric Surgery, Juntendo University Nerima Hospital, Tokyo, Japan; ^3^Department of Pathology, Shin-Yurigaoka General Hospital, Kawasaki, Japan; ^4^Department of Pathology, Tohoku University Graduate School of Medicine, Sendai, Japan

## Abstract

Dysgerminoma is a malignant ovarian germ cell tumor, and unlike sex-cord stromal tumors, endocrine manifestation is considered rare. Here, we report the first case of dysgerminoma presenting precocious puberty. The patient is a 7-year-old girl who presented with a breast development in Tanner stage 3. Serum estradiol (E_2_) was markedly elevated while luteinizing hormone (LH) and follicle-stimulating hormone (FSH) were suppressed below the detection limit. Microscopically, the right ovarian mass displayed nests of large polygonal cells and fibrous septa which were focally concentrated by theca-like plump spindle cells. Immunohistochemistry revealed that the spindle cells expressed various steroidogenic enzymes involved in estrogen biosynthesis including P450 aromatase. The tumor was diagnosed with pure dysgerminoma with estrogen-producing functioning stroma. After the operation, serum E_2_ declined below the detection limit; LH and FSH returned within the normal range. This case demonstrates that even a conventional dysgerminoma can present endocrine manifestation through functioning stroma.

## 1. Introduction

Dysgerminoma is a malignant ovarian germ cell tumor, and unlike sex cord-stromal tumors, endocrine manifestation is considered rare [1]. To date, three cases of dysgerminoma with estrogenic manifestation have been reported all of which presented an unusual morphology that contained a human chorionic gonadotropin- (hCG-) secreting syncytiotrophoblast [2–4]. It has been postulated that hCG transformed nonneoplastic ovarian stroma, the so-called functioning stroma, and produced estrogen. However, endocrine manifestation in a conventional dysgerminoma that lacks a syncytiotrophoblast has never been reported. Here, we report the first case of dysgerminoma with hyperestrogenism that showed a conventional morphology.

## 2. Case Presentation

A 7-year-old girl presented with a breast development with four-month duration, vaginal discharge, and rapid height increase. Her past medical history and family history were unremarkable. She was 130 cm in height (+2.24 standard deviation) and 28.9 kg in weight (+1.62 standard deviation). Physical examination revealed a breast development in Tanner stage 3, but pubic and axillary hair was not observed. Abdominal ultrasonography revealed a solid mass at the right side of the rectouterine pouch. Serum estradiol (E_2_) level was 41.7 pg/mL and markedly elevated for her age while luteinizing hormone (LH) and follicle-stimulating hormone (FSH) were suppressed below the detection limit (Table 1). An estrogen-producing ovarian tumor was suspected. The patient underwent laparoscopic right salpingo-oophorectomy.

**Figure 1:**
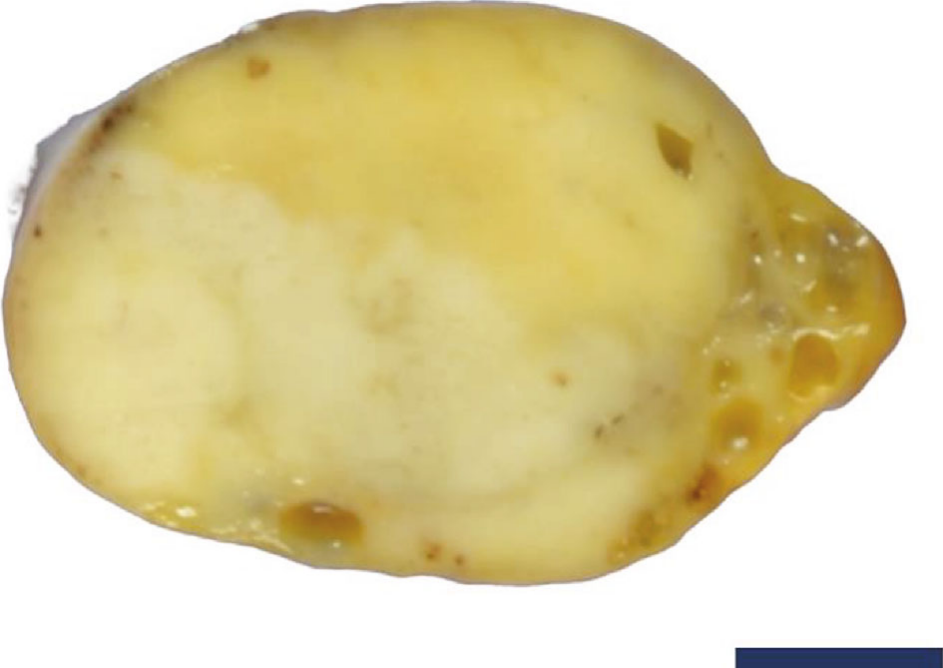
Gross examination of the right ovary exhibiting a solid and fleshy mass with a tan-yellow cut surface. Scale bar indicates 1 cm.

**Figure 2:**
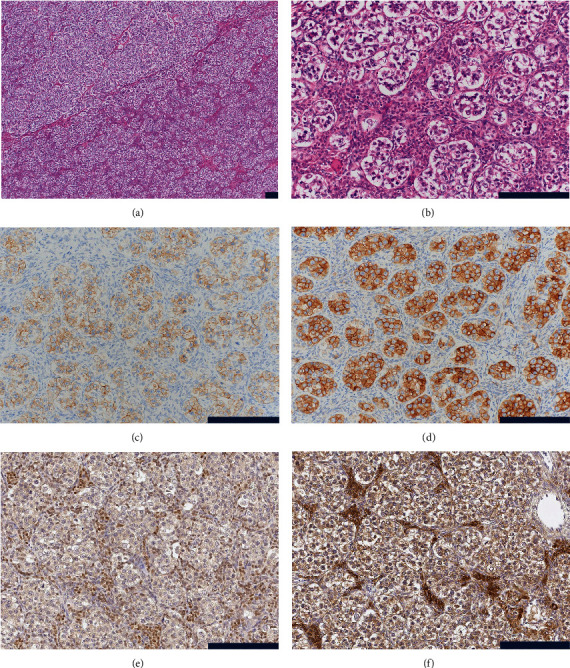
Nests of tumor cells intersected by fibrous septa (a). The septa were concentrated by theca-like plump spindle cells (b). Tumor cells were positive for c-kit (c) and podoplanin (d). Stromal cells were positive for SF-1 (e) and P450_arom_ (f). Scale bars indicate 200 *μ*m.

On gross examination, the right ovary was occupied by a 49 × 40 × 26 mm solid and fleshy mass with a tan-yellow cut surface (Figure 1). Microscopically, the mass showed sheets and nests of large polygonal cells intersected by fibrous septa (Figure 2(a)). The septa were concentrated by stromal cells with plump spindle nuclei that resembled theca cells (Figure 2(b)). The tumor cells displayed abundant clear cytoplasm with large round nuclei and conspicuous nucleoli. They also expressed placental alkaline phosphatase, c-kit (Figure 2(c)), and podoplanin (Figure 2(d)), and thus, the tumor was diagnosed with pure dysgerminoma. A typical *two-cell pattern* was not recognized, but immunohistochemistry for CD3 revealed a few T lymphocytes scattered within the nests.

Further investigation was performed to immunolocalize the steroidogenic enzymes. Stromal cells were positive for steroidogenic factor 1 (SF-1) (Figure 2(e)), P450_C17_ (17*α*-hydroxylase/17,20-lyase), 3*β*-hydroxysteroid dehydrogenase (HSD), P450_arom_ (aromatase) (Figure 2(f)), 17*β*-HSD type 1, and steroid sulfatase but negative for 17*β*-HSD types 2 and 5, 5*α*-reductases 1 and 2, and estrone sulfotransferase. These results indicated that the stromal cells were enzymatically active and produced E_2_ independently.

The postoperative course was uneventful, and the patient was discharged three days after the surgery. Serum E_2_ level declined below the detection limit; LH and FSH returned within the normal range. The serum *β*-hCG level was constantly low throughout the observation (Table 1). The patient underwent three courses of postoperative chemotherapy, and no sign of recurrence has been observed for at least two years.

## 3. Discussion

We reported a case of dysgerminoma with estrogen-producing functioning stroma. Although three similar cases have been reported to date, the mechanism of estrogen biosynthesis in our case was distinct from the previous cases [2–4]. Functioning stroma is a nonneoplastic ovarian stroma that produces various sex steroids. It is commonly observed in mucinous cystadenomas and Krukenberg tumors, but it can arise in any type of ovarian tumor [5, 6]. The development of functioning stroma remains unclear; however, since it is frequently observed in pregnant women, hCG is regarded as one of the triggers [5, 6]. All previous cases of dysgerminoma with hyperestrogenism presented elevated serum hCG levels and histology of a special subtype that contains abundant syncytiotrophoblast. Thus, it has been postulated that the syncytiotrophoblast produced hCG, as in normal placenta, and induced functioning stroma [5, 7]. On the contrary, our case lacked syncytiotrophoblast, and the serum hCG level was constantly low throughout the observation. However, we discovered that the stromal cells expressed various steroidogenic enzymes involved in estrogen biosynthesis and produced estrogen independently.

Among these enzymes, P450_arom_ is the most important factor as it catalyzes the final step of estrogen biosynthesis. Nevertheless, since it is widely distributed in the peripheral tissue, determining how much the functioning stroma contributes to aromatization is difficult. Previous studies reported that 9 to 80% of the ovarian tumors with functioning stroma expressed P450_arom_; however, the authors conceived that most aromatization may have occurred in the peripheral tissue rather than the ovary as the expression of P450_arom_ was focal [5, 8]. In our case, the stromal cells were diffusely positive for P450_arom_ suggesting that most stroma-derived androgen were aromatized by the stromal cells in an autocrine manner.

Our case demonstrates that even a conventional dysgerminoma can produce estrogen through functioning stroma. Since most dysgerminomas occur during the reproductive age where endogenous estrogen can mask hyperestrogenism [1, 6], many estrogen-producing dysgerminomas may have been overlooked. This presumption is also supported by the fact that endocrine manifestation has only been reported in prepubertal patients. Although serum hormone levels are not measured routinely in ovarian tumors, immunohistochemistry for the steroidogenic enzymes, especially P450_arom_, could be utilized in the future study to reveal subclinical endocrine manifestation in dysgerminoma.

## Figures and Tables

**Table 1:** Changes of serum hormone levels before (pre-op) and after (post-op) operation.

	Pre-op	Post-op (POD 0)	Post-op (POD 8)
E_2_ (pg/mL)	41.7	8.8	b.d.l.
LH (mIU/mL)	b.d.l.	b.d.l.	0.9
FSH (mIU/mL)	b.d.l.	b.d.l.	3.6
*β*-hCG (ng/mL)	0.2	0.2	0.2

b.d.l.: below the detection limit; E_2_: estradiol; FSH: follicle-stimulating hormone; LH: luteinizing hormone; POD: postoperative days; *β*-hCG: human chorionic gonadotropin beta subunit.
